# PCNA tightens its hold on the nucleus

**DOI:** 10.1080/15384101.2015.1064699

**Published:** 2015-07-15

**Authors:** Lynne S Cox

**Affiliations:** Department of Biochemistry; University of Oxford; Oxford, UK

The sliding clamp protein PCNA is a key controller of multiple processes in DNA and chromatin metabolism, regulating replication, repair and chromatin assembly through interaction with a huge number of partner proteins (**Fig 1**). In many of these transactions, the partners bind via a conserved PCNA-interacting peptide or PIP, first identified in CDKN1/p21.^1^ Sequential and competitive binding of these PIPs within the interdomain connector loop (IDCL) of PCNA provides a means to ensure ordered reactions.^2,3^

**Figure 1.**
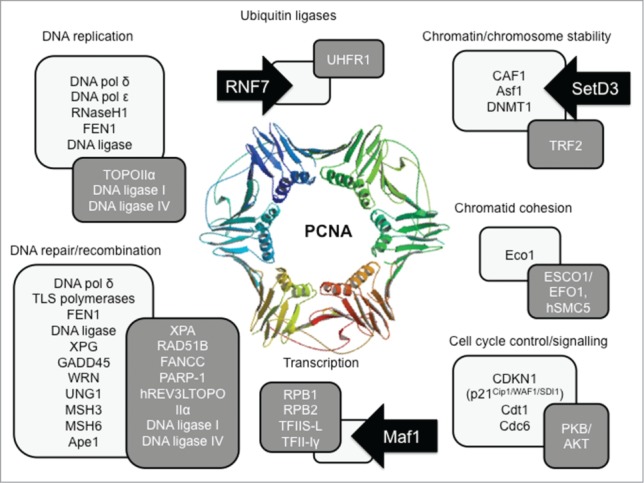
PCNA's many partners. Interactions with PCNA (structure from PDB 1axc) can be through classical PIP motif (Qxx[L/I/M]xx[F/Y][F/Y],^1,2^ (pale boxes)) binding to the interdomain connector loop in PCNA or via an APIM motif ([K/R]VF[I/V]K)^5^ (dark boxes). The newly identified PCNA partners Maf1, RNF7 and SetD3^4^ (black arrows) contribute to transcription, ubiquitination and chromatin regulation.

Adding to the roll call of PCNA partners, Cooper et al.^4^ have now employed bimolecular fluorescence complementation (BiFC) screening to look for proteins that bind to PCNA ‘bait’ in normally proliferating human cells; unlike *in vitro* interaction studies and heterologous yeast 2 hybrid screens, BiFC screening in cycling human cells provides a platform for physiologically relevant protein interaction discovery. Combining FACS sorting with the speed and depth of reads possible with next generation sequencing also allows the scale-up of this type of screen for identification of drug targets. Notably, this screen^4^ identified novel PCNA partners that do not necessarily interact through the canonical PIP (though a number of the expected partners were also detected). Interactions were not universal across all cells – perhaps because the initial screen did not enrich for S phase cells – nor particularly robust (the signal was lost on detergent treatment), while use of a skeletal muscle cDNA library probably influenced the range of partner proteins identified – SetD3, for instance, is important in muscle differentiation. Nevertheless, this screen is powerful and interactions with RNF7, Maf1 and SetD3 were validated by a range of direct assays.

So what might these novel partners tell us about how PCNA acts in cells? SetD3 is a histone H3 lysine methyltransferase that adjusts the histone code to promote a transcription-competent chromatin conformation. Perhaps interaction here simply adds to PCNA's repertoire in assisting copying of the histone code immediately after replication fork passage, as postulated from its association with Caf1 and Asf1. While SetD3 does not have every residue of a classical PIP, the motif QKGLSVTF may be adequate for association with PCNA's IDCL. Notably the aromatic residues play a critical role in binding affinity with tyrosine (e.g. p21 PIP = QTSMTDF**Y**) conferring much tighter PCNA binding than phenylalanine (e.g., Fen1 PIP= QGRLDDF**F**). Maf1 and RNF7 are small proteins without identifiable PIPs; but lack of a PIP does not prevent other partners such as RF-C from binding PCNA with high affinity, nor proteins that bind through the alternative APIM motif.^5^

Regulation of the cell cycle through PIP-dependent degradation of key protein such as p27, Cdt1 and – most recently - Cdc6^6^ provides yet another critical role for PCNA. Its interaction with RNF-7, an essential Skp1-cullin/cdc53 F box ubiquitin ligase is therefore not unexpected, and may help to ensure that replication occurs once and only once per cell cycle. Given that MCM disassembly from terminating replication forks requires MCM7 ubiquitination by an as yet unidentified cullin-family protein,^7^ PCNA at a terminating replication fork might also provide a platform for recruitment of an ubiquitin ligase at the right time and place to regulate replisome disassembly. Integration of nutritional status and stress signals is essential to ensure appropriate cell cycle progression; increasing evidence supports a cytosolic role for PCNA in signaling, with APIM peptide-containing proteins implicated in cell cycle control and damage signaling.^5^ Hence the finding that Maf1, a transcriptional repressor, binds PCNA might provide a further link between cytosolic signaling (in this case via mTORC, which targets Maf1) and transcriptional responses. PIP or no PIP, PCNA partners play a huge role in the cell and PCNA is turning out to be one of the major hubs coordinating cellular metabolism.

## References

[1] Warbrick E, et al. Curr Biol 1995; 5(3):275-82; PMID:7780738; http://dx.doi.org/10.1016/S0960-9822(95)00058-3 7780738

[2] Cox LS. Trends Cell Biol 1997; 7(12):493-8; PMID:17709013; http://dx.doi.org/10.1016/S0962-8924(97)01170-7 17709013

[3] Dovrat D, et al. Proc Natl Acad Sci U S A 2014; 111(39):14118-23; PMID:25228764; http://dx.doi.org/10.1073/pnas.1321349111 25228764 PMC4191785

[4] Cooper SE, et al. Cell Cycle. 2015 [Epub ahead of print]; PMID:26030842 26030842 10.1080/15384101.2015.1053667 PMC4613188

[5] Gilljam KM, et al. J Cell Biol 2009; 186(5):645-54; PMID:19736315 19736315 10.1083/jcb.200903138 PMC2742182

[6] Clijsters L, Wolthuis R. J Cell Sci 2014; 127(6):1336-45; PMID:24434580; http://dx.doi.org/10.1242/jcs.145862 24434580

[7] Moreno SP, et al. Science 2014; 346(6208):477-81; PMID:25342805; http://dx.doi.org/10.1126/science.1253585 25342805

